# Persistence of Severe Acute Respiratory Syndrome Coronavirus 2 in Aerosol Suspensions

**DOI:** 10.3201/eid2609.201806

**Published:** 2020-09

**Authors:** Alyssa C. Fears, William B. Klimstra, Paul Duprex, Amy Hartman, Scott C. Weaver, Kenneth S. Plante, Divya Mirchandani, Jessica Ann Plante, Patricia V. Aguilar, Diana Fernández, Aysegul Nalca, Aysegul Totura, David Dyer, Brian Kearney, Matthew Lackemeyer, J. Kyle Bohannon, Reed Johnson, Robert F. Garry, Doug S. Reed, Chad J. Roy

**Affiliations:** Tulane University School of Medicine, New Orleans, Louisiana, USA (A.C. Fears, R.F. Garry, C.J. Roy):; University of Pittsburgh, Pittsburgh, Pennsylvania, USA (W.B. Klimstra, P. Duprex, A. Hartman, D.S. Reed);; University of Texas Medical Branch, Galveston, Texas, USA (S.C. Weaver, K.S. Plante, D. Mirchandani, J.A. Plante, P.V. Aguilar, D. Fernández);; U.S. Army Medical Research Institute of Infectious Diseases, Fort Detrick, Maryland, USA (A. Nalca, A. Totura, D. Dyer, B. Kearney);; National Institute of Allergy and Infectious Diseases, National Institutes of Health, Fort Detrick, Maryland, USA (M. Lackemeyer, J.K. Bohannon, R. Johnson);

**Keywords:** aerosol, coronavirus diseases, 2019 novel coronavirus disease, COVID-19, SARS-CoV-2, severe acute respiratory syndrome coronavirus 2, respiratory diseases, zoonoses, viruses, Middle East respiratory syndrome coronavirus, MERS-CoV, severe acute respiratory syndrome coronavirus, SARS-CoV

## Abstract

We aerosolized severe acute respiratory syndrome coronavirus 2 and determined that its dynamic aerosol efficiency surpassed those of severe acute respiratory syndrome coronavirus and Middle East respiratory syndrome. Although we performed experiment only once across several laboratories, our findings suggest retained infectivity and virion integrity for up to 16 hours in respirable-sized aerosols.

Severe acute respiratory syndrome coronavirus 2 (SARS-CoV-2), is a readily transmissible zoonotic pathogen and the etiologic agent of the coronavirus disease (COVID-19) pandemic (*1*). To determine aerosol stability of the virus, we measured the dynamic (short-term) aerosol efficiencies of SARS-CoV-2 and compared its efficiency with SARS-CoV and Middle East respiratory syndrome coronavirus (MERS-CoV).

## The Study

We analyzed these 3 viruses’ dynamic aerosol efficiencies using 3 nebulizers: the Collison 3-jet (C3), Collison 6-jet (C6) (www.chtechusa.com), and Aerogen Solo (AS) (https://www.aerogen.com), to generate viral aerosols (Appendix). We performed comparative efficiency experiments once in each of 4 aerobiology laboratories (Tulane University, New Orleans, LA, USA; National Institutes of Health Integrated Research Facility [NIH-IRF], Fort Detrick, MD, USA; US Army Medical Institute for Infectious Diseases, Fort Detrick, MD, USA; and University of Pittsburgh, Pittsburgh, PA, USA). The aerosol size distributions produced by the generators used, in mass median aerodynamic diameter, were 1–3 μm and had a geometric heterodispersity of ≈1.2–1.4. Aerosols were generated into 16-liter primate head-only exposure chambers (MERS-CoV or SARS-CoV-2) or a 30-liter rodent chamber (SARS-CoV), where the overall flow was ≈1 (Tulane University) or 0.5 (NIH-IRF, US Army Medical Research Institute of Infectious Diseases, University of Pittsburgh) air changes per minute. Use chamber and corresponding flow rates enabled us to determine the dynamic efficiencies of the virus in aerosols during a short residence time. Samples were continuously collected and integrated throughout the initiation of respective nebulizers into the chamber during aerosol generation events of 10–30 min. We calculated the dynamic aerosol efficiency or spray factor (*Fs*) as a unitless quotient of initial titer (PFU/L in liquid stock) to the resulting aerosol (PFU/L aerosol) providing a quantitative indicator for comparing airborne fitness (*2*,*3*).

**Figure 1:**
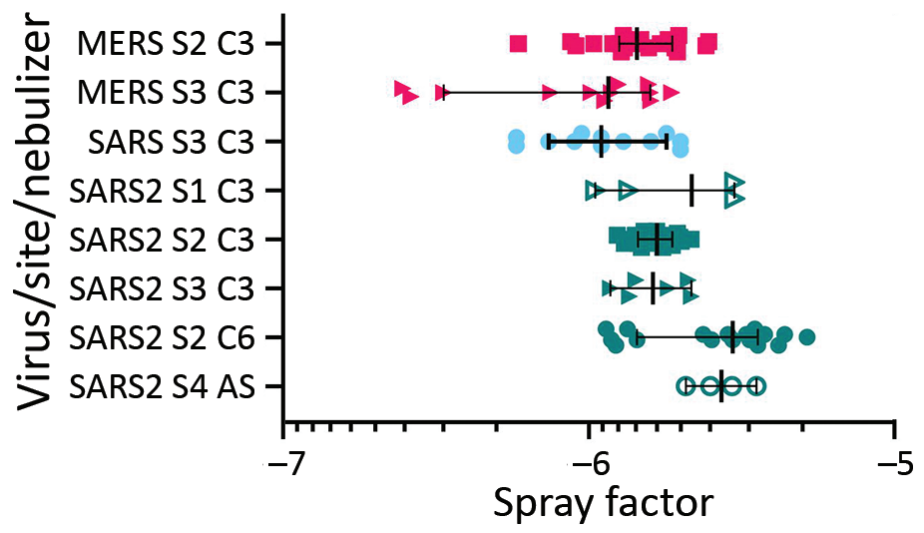
Aerosol efficiency of MERS-CoV, SARS-CoV and SARS-CoV-2 at different sites. Graph shows the spray factor (i.e., ratio of nebulizer concentration to aerosol concentration) for MERS-CoV (red), SARS-CoV (blue), and SARS-CoV2 (green). Aerosols were performed at 4 sites and with different nebulizers. AS, Aerogen Solo nebulizer; C3, Collison 3-jet nebulizer; C6, Collison 6-jet nebulizer; MERS-Cov, Middle East respiratory syndrome coronavirus; S1, Tulane University, New Orleans, LA, USA; S2, National Institutes of Health Integrated Research Facility, Fort Detrick, MD, USA; S3, US Army Medical Institute for Infectious Diseases, Fort Detrick, MD, USA; S4, University of Pittsburgh, Pittsburgh, PA, USA; SARS-CoV, severe acute respiratory syndrome coronavirus; SARS-CoV-2, severe acute respiratory syndrome coronavirus 2.

We determined *Fs* for all 3 viruses after <1 min of chamber residence after aerosolization (Figure 1). When we compared both MERS-CoV and SARS-CoV to SARS-CoV-2 aerosols generated with a C3 nebulizer across 3 laboratories, we noted a small but significant improvement in *Fs* for SARS-CoV-2 but not for SARS-CoV (p = 0.02) or MERS-CoV (p = 0.01). Because SARS-CoV was aerosolized into a different chamber/volume than MERS-CoV and SARS-CoV-2, we cannot rule out chamber effects for the difference in *Fs* between SARS-CoV and SARS-CoV-2. Our comparison of nebulizers showed improved *Fs* for SARS-CoV-2 with the C6 (p = 0.006) and the AS (p = 0.01) over the C3 but no difference between the C6 and AS (p = 0.46).

Further studies with SARS-CoV-2 at Tulane University preliminarily assessed the long-term stability of airborne virus. We used a custom-built rotating (Goldberg) drum to provide an environment in which rotational drum speed overcomes the terminal settling velocity of the 2–3-μm particles, thereby providing a static aerosol suspension of known volume (*4*–*6*). We timed aerosol samples from the drum at 10 min and 30 min and at 2 h, 4 h, and 16 h after initiation of rotation/suspension. The entire drum volume (10.7 L) was evacuated at each sampling interval and represented a discrete aerosol generation event. We quantified virus contents by plaque assay and reverse transcription quantitative PCR (RT-qPCR). We also conducted scanning electron microscopy on the collected aerosol samples as a complimentary qualitative assessment of virion integrity after longer-term aerosol suspension (Appendix). We measured environmental parameters but did not control them during the aerosol suspension experiments. The prevailing ambient environmental conditions were 23°C ± SD 2°C and 53% ± SD 11% relative humidity throughout the aerosol stability experiments. No ultraviolet light source was used within the cavity of the drum during suspensions. After initial generation of viral bioaerosols into the drum reached steady-state concentration, the drum was sealed and maintained as a static aerosol. We conducted all sampling time points once in this set of experiments.

**Figure 2:**
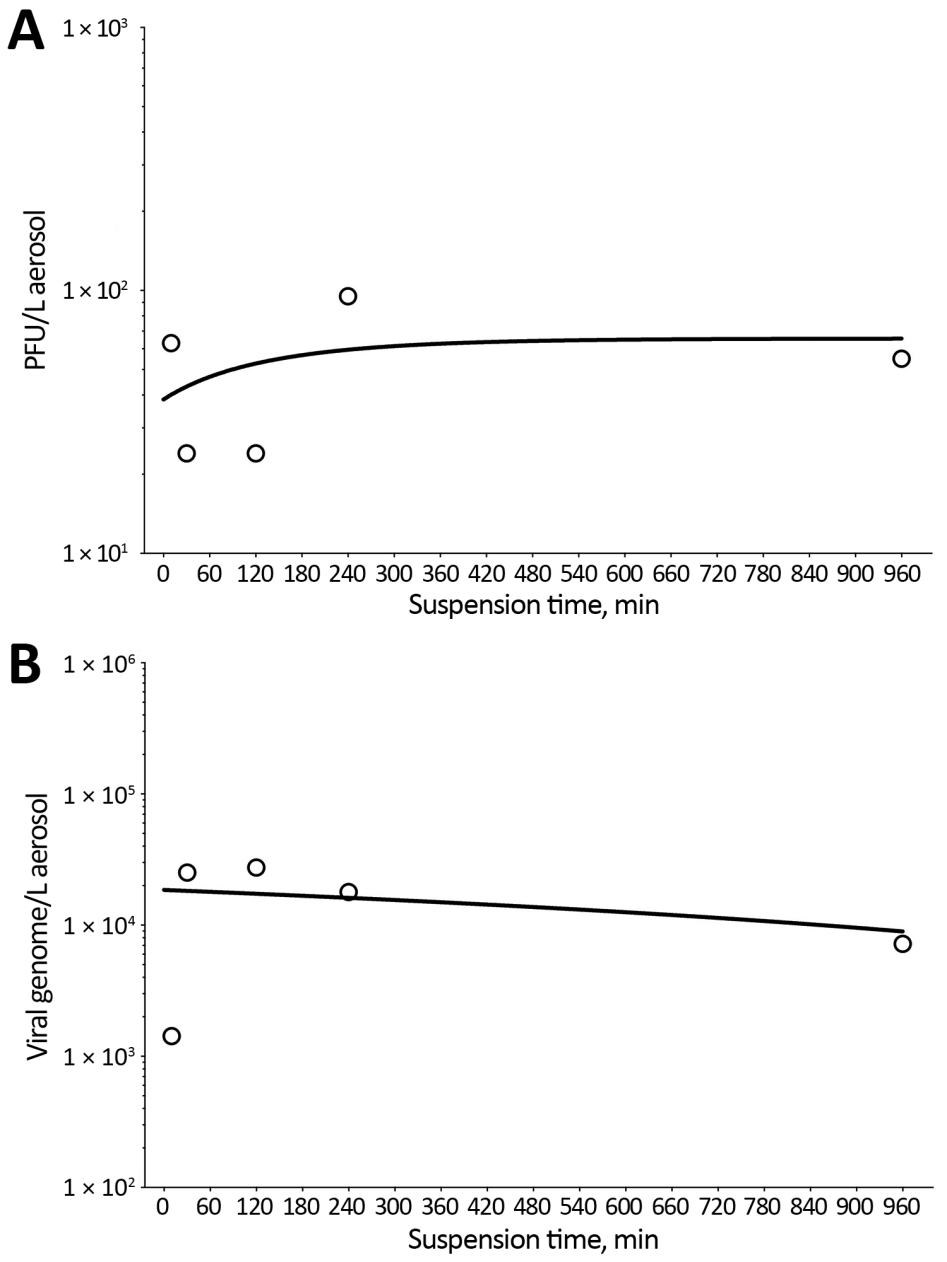
Decay curves of severe acute respiratory syndrome coronavirus 2 (SARS-CoV-2) in aerosol suspension. A) Aerosol concentration of infectious SARS-CoV-2 as measured by plaque assay found in impinger samples collected at 5 time points of increased aging in aerosol suspension. B) Corresponding aerosol concentration of SARS-CoV-2 in time-matched impinger samples as a function of viral genome copies as measured by reverse transcription quantitative PCR. Both time point virus estimates were graphed, and nonlinear least-squares regression analysis single-order decay with no outlier detection was performed, resulting in a poor curve fit by either method of viral quantitation resulting from number and lack of iterative samples in this analysis.

We graphed plaque assay and RT-qPCR results and applied nonlinear least-squares regression analysis single-order decay with no outlier detection, resulting in a poor curve fit, which typically results from a lack of replicate samples. We detected infectious SARS-CoV-2 at all time points during the aerosol suspension stability experiment (Figure 2). A minor but constant fraction of SARS-CoV-2 maintained replication-competence at all time points (Figure 2, panel A), including when sampled after 16 h of aerosol suspension. This finding resulted in a remarkably flat decay curve when measured for infectivity and failed to provide a biologic half-life (κ = 2.93E-06; t_1/2_ = 2.36E+05; τ = 3.40E+05). The curve (Figure 2, panel B) from the results of split sample analysis as quantified by RT-qPCR showed minimal decreases in aerosol concentration measured in viral genome copies across all of time points sampled and approximated the decay curve of the infectious virus fraction (Figure 2, panel A), including similar decay curve characteristics (κ = 6.19E-03; t_1/2_ = 111.9; τ = 161.4).

We also performed a qualitative assessment of virion integrity after longer-term aerosol suspension (Appendix). Scanning electron microscopy (SEM) imaging of SARS-CoV-2 revealed virions that were heterogeneous in shape, either ovoid (Appendix Figure, panel A) or spherical (Appendix Figure, panel B). The minor:major axis ratio of oval virions was ≈0.7, which is consistent with prior SEM analyses of SARS-CoV-2 (https://www.flickr.com/photos/niaid/albums/72157712914621487). Airborne SARS-CoV-2 maintained the expected morphologies, size, and aspect ratios up to 16 h. Specifically, virions aged for 10 min (Appendix Figure, panels C, D) or 16 h (Appendix Figures, panels E, F) were similar in shape and general appearance to virions examined in samples of viral inoculum collected before aerosolization, which is consistent with the retention of replication-competence and suggests the potential to be infectious after long-term aging in aerosol suspension.

## Conclusions

The comparison of short-term aerosol efficiencies of 3 coronaviruses showed SARS-CoV-2 approximates or exceeds the efficiency estimates of SARS-CoV and MERS-CoV. Some efficiency determinations for SARS-CoV-2 ranged to −5.5^log10^ (Figure 1), a full log difference from MERS-CoV. The higher efficiencies across independent laboratories strengthens this observation. These data suggest that SARS-CoV-2 generally maintains infectivity at a respirable particle size over short distances, in contrast to either betacoronavirus. Aerosol suspension results suggest that SARS-CoV-2 persists longer than would be expected when generated as this size particle (2-µm mass median aerodynamic diameter). This finding is notable because decay and loss in the infectious fraction of airborne virus would be expected on the basis of prior susceptibility studies with other environmentally hardy viruses, such as monkeypox virus (*5*). A recent study (*6*) showing only a slight reduction of infectivity in aerosol suspensions with approximately similar particle sizes also suggested minimal effects on SARS-CoV-2 airborne degradation.

Collectively, these preliminary data suggest that SARS-CoV-2 is resilient in aerosol form and agree with conclusions reached in earlier studies of aerosol fitness (*6*). A clear limitation of the aerosol stability data is that we report only 1 measurement of the 16-h time point; future studies need to repeat these findings before any definitive conclusions are reached. Aerosol transmission of SARS-CoV-2 may be a more important exposure transmission pathway than previously considered (*7*). Our approach of quantitative measurement of infectivity of viral airborne efficiency augmented by assessment of virion morphology suggests that SARS-CoV-2 may be viable as an airborne pathogen. Humans produce aerosols continuously through normal respiration (*8*). Aerosol production increases during respiratory illnesses (*9*,*10*) and during louder-than-normal oration (*11*). A fraction of naturally generated aerosols falls within the size distribution used in our experimental studies (<5 μm), which leads us to conclude that SARS-CoV-2–infected persons may produce viral bioaerosols that remain infectious for long periods after production through human shedding and airborne transport. Accordingly, our study results provide a preliminary basis for broader recognition of the unique aerobiology of SARS-CoV-2, which might lead to tractable solutions and prevention interventions.

Appendix Additional methods and results in a study of aerosol efficiencies of coronaviruses.
