# Supplementary material for: Persistence of Severe Acute Respiratory Syndrome Coronavirus 2 in Aerosol Suspensions
Source: Emerg Infect Dis. 2020 Sep;26(9):2168–71. doi: 10.3201/eid2609.201806 (PMC7454081; doi:10.3201/eid2609.201806)
Supplement: Appendix — Additional methods and results in a study of aerosol efficiencies of coronaviruses. [file 20-1806-Techapp-s1.pdf]

Article DOI: https://doi.org/10.3201/eid2609.201806

Persistence of Severe Acute Respiratory Syndrome Coronavirus 2 in Aerosol Suspensions

Appendix

Methods

Viruses

The viruses used for the comparative studies included SARS-CoV-2; 2019-nCoV/USA-WA1/2020 (MN985325.1), SARS-CoV-2/Munchen-1.1/2020/929; SARS-CoV, Urbani; and MERS-CoV-hNIS, and MERS-CoV Hu/Jordan-N3/2012 (KJ614529); EMC/2012 (JX869059.2). Virus stocks were prepared in Vero E6 cells and sequence confirmed by PCR and/or Sanger sequencing. Plaque assays were performed in Vero E6 cells. A manuscript is in preparation with a more detailed description of virus propagation and plaque assays (Klimstra, W., Duprex, P.).

Dynamic Aerosol Exposures

Aerosol exposures were conducted using the Automated Bioaerosol Exposure System (ABES) or AeroMP (Biaera Technologies, Hagerstown, MD, USA) exposure management platform inside a Class III biologic safety cabinet operated under negative pressure. The 3-jet (C3), 6-jet Collison (C6), or Aerogen Solo (AS) nebulizers were used for generation of aerosols. Aerosol sampling was performed with an all-glass impinger (AGI)-4 (Ace Glass, Vineland, NJ, USA) or SKC Biosampler (SKC Inc, Eighty Four, PA, USA). Aerosol particle size was determined using an Aerodynamic Particle Sizer solely or with the addition of a diluter (TSi, Shoreview, MN, USA).

SARS-CoV-2 Quantitative Real-Time Reverse Transcription PCR

Extracted nucleic acid samples were tested for SARS-CoV-2 by RT-qPCR following a previously published assay (*1*). Two RT-qPCR monoplexes targeting the N and ORF1b were used. The primers and probes used to amplify the ORF1b gene are: Forward primer [5′-TGGGGYTTTACRGGTAACCT-3′]; reverse primer [5′-AACRCGCTTAACAAAGCACTC-3′];

and probe [5′-FAM-TAGTTGTGATGCWATCATGACTAG-TAMRA-3′]. The N gene was amplified using the following primers and probe: Forward primer [5′-TAATCAGACAAGGAACTGATTA-3′]; reverse primer [5′-CGAAGGTGTGACTTCCATG-3′]; and probe [5′-FAM-GCAAATTGTGCAATTTGCGG-TAMRA-3′]. Briefly, a 20-μL reaction mixture was prepared using the iTaq Universal probes One-Step kit (BioRad, Hercules, CA, USA), according to manufacturer instructions: 10 μL of reaction mix (2×), 0.5 μL of iScript reverse transcription, 1 μL primers (10 μM), 0.5 μL probes (10 μM), 4 μL of extracted RNA and 3 μL of water. The RT-qPCR reactions were conducted using the thermocycler StepOnePlus Real-Time PCR Systems (Applied Biosystems). Reactions were incubated at 50°C for 5 min and 95°C for 20 sec followed by 40 cycles at 95°C for 5 sec and 60°C for 30 sec. Serial dilutions of the RNA extracted from a virus stock with known concentration was included for generation of a standard curve and to estimate the amount of targets in the experimental samples.

Scanning Electron Microscopy

Five mm × 2 mm hydroxyapatite (HAp) discs (Clarkson Chromatography) were incubated with 750 μL of each virus sample in DMEM media within a 24-well plate for 30 min. Sterile forceps were used to transfer each disc to 2.5% glutaraldehyde in deionized water for 12 h. Samples were further fixed in 1% osmium (VIII) oxide in 100 mM PBS for 30 min then rinsed in deionized water 3x. Samples were prepared for scanning electron microscopy by drying in successive increasing concentrations of ethyl alcohol (from 25%–100%) each for 10 min and critical point dry in a Tousimis® Autosamdri-814 dryer before mounting with carbon tape, double carbon sputter-coating in a Cressington® 208HR sputter coater, and imaging at 3 kV in a Hitachi S-3400 SEM

Analysis

Significance determined in Figure 1 by Mann-Whitney and Brown-Forsythe tests and nonlinear regression analysis in Figure 2 performed in GraphPad Prism version 8.4.1.

Reference

1. Roy CJ, Reed DS. Infectious disease aerobiology: miasma incarnate. Front Cell Infect Microbiol. 2012;2:163. PubMed https://doi.org/10.3389/fcimb.2012.00163

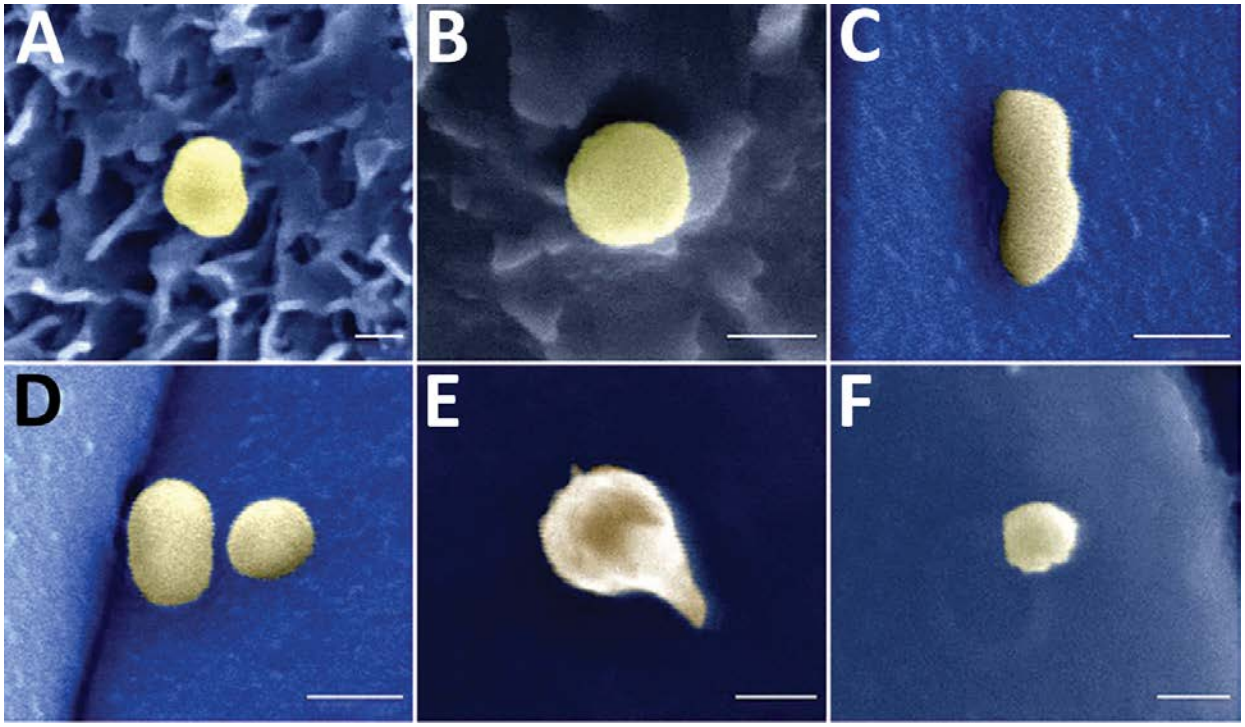

Appendix Figure. Electron microscopy images of severe acute respiratory syndrome coronavirus 2 in aerosol suspension at various timepoints. A, B) From viral stock before aerosolization. C, D) From 10-minute aerosol suspension. E, F) From 16-hour aerosol suspension. Scale bars = 100 nm.
